# Nosocomial bloodstream infections caused by *Klebsiella pneumoniae*: impact of extended-spectrum β-lactamase (ESBL) production on clinical outcome in a hospital with high ESBL prevalence

**DOI:** 10.1186/1471-2334-6-24

**Published:** 2006-02-14

**Authors:** Alexandre R Marra, Sérgio B Wey, Adauto Castelo, Ana Cristina Gales, Ruy Guilherme R Cal, José R do Carmo Filho, Michael B Edmond, Carlos Alberto P Pereira

**Affiliations:** 1 Division of Infectious Diseases, Universidade Federal de São Paulo, Brasil (UNIFESP-EPM)/Hospital São Paulo (HSP), Brasil; 2 Clinical Microbiology Laboratory, Universidade Federal de São Paulo, Brasil (UNIFESP-EPM)/Hospital São Paulo (HSP), Brasil; 3 Intensive Care Unit, Hospital Israelita Albert Einstein, São Paulo, Brasil; 4 Department of Internal Medicine, Medical College of Virginia Campus, Virginia Commonwealth University, Richmond, Virginia, USA

## Abstract

**Background:**

The frequency of ESBL producing *Klebsiella pneumoniae *bloodstream infections (BSI) is high in Brazilian hospitals, however little is known regarding what role, if any, resistance plays in the expected outcome in hospitals with a high prevalence of these pathogens.

**Methods:**

From 1996 to 2001, hospital acquired *K. pneumoniae *BSI were evaluated retrospectively. Each patient was included only once at the time of BSI. ESBL producing strains were identified using the E-test method. The association of variables with the mortality related to bacteremia was included in a stepwise logistic regression model.

**Results:**

One hundred and eight hospital acquired *K. pneumoniae *BSI met criteria for inclusion. Fifty two percent were due to ESBL producing strains. The overall in-hospital mortality was 40.8%. Variables independently predicting death by multivariate analysis were the following: mechanical ventilation (**p = 0.001**), number of comorbidities (**p = 0.003**), antimicrobials prescribed before bacteremia (**p = 0.01**) and fatal underlying disease (**p = 0.025**).

**Conclusion:**

Bacteremia due to ESBL producing *K. pneumoniae *strains was not an independent predictor for death in patients with BSI. An increased mortality in hospital-acquired BSI by *K. pneumoniae *was related to the requirement for mechanical ventilation, more than two comorbidities, the previous use of two or more antibiotics, and the presence of a rapidly fatal disease.

## Background

*Klebsiella pneumoniae *is an important cause of many infections [1]. It ranks among the top ten pathogens that cause bloodstream infection (BSI) in the United States and Canada [2]. In Latin America, it is the third most prevalent pathogen isolated in the respiratory tract of hospitalized patients with pneumonia and corresponds to 12% of all pathogens isolated [3].

Extended-spectrum β-lactamase (ESBL) producing organisms were first isolated in Germany in 1983 [4] and in the United States in 1989 [5]. ESBLs are plasmid-mediated enzymes that hydrolyze broad-spectrum β-lactams [6]. The emergence of ESBL-producing *K. pneumoniae *has been reported as an important cause of nosocomial infection in the United States and Europe. The prevalence of ESBL-producing *K. pneumoniae *strains in hospitals, ranges from 5 to 25% in several parts of the world [7,8]. In Brazilian hospitals, the frequency of ESBL-producing *K. pneumoniae *is higher than those observed in many European and American hospitals, accounting for 45% of *K. pneumoniae *strains [3].

It has been shown that a poor outcome occurs when patients with serious infections due to ESBL-producing organisms are treated with antibiotics to which the organism is highly resistant [9]. The mortality rate in such patients is significantly higher than those observed in patients treated with antibiotics to which the organism is susceptible. In addition, a suboptimal clinical outcome occurs when cephalosporins are used for the treatment of serious infections due to ESBL-producing organisms, which may appear to be susceptible on the basis of cephalosporin MICs of 2 to 8 μg/mL [7].

Risk factors associated with infections caused by ESBL producing organisms include central venous catheters [10], tracheostomy [11], and cephalosporin use [11], however little is known regarding what role, if any, resistance plays in the expected outcome in hospitals with a high prevalence of these pathogens.

The aim of this study was to evaluate whether ESBL producing *K. pneumoniae *is associated with a high mortality rate in a hospital with a high ESBL prevalence.

## Methods

A retrospective cohort study was carried out at the Universidade Federal de São Paulo, a 624-bed university hospital, located in the state of São Paulo, Brazil. The study was approved by the Hospital Ethics Committee. All patients for whom blood culture results were positive for *K. pneumoniae *from January 1996 to May 2001 were eligible for inclusion in the study. Each patient was included only once. If multiple blood cultures from the same patient were positive for the above organism, only the first episode was reviewed and recorded. Pseudobacteremia caused by *K. pneumoniae*, defined as the presence of a positive blood culture without clinical manifestations of sepsis (fever, hypotension, tachycardia, tachypnea and leukocytosis or leukopenia), and cases with incomplete data in the medical record were excluded.

### Clinical and laboratory characteristics of patients

Potential risk factors for mortality due to *K. pneumoniae *infection were ascertained by means of a review of medical charts. Data obtained included age, sex, ward, number of hospital days prior to infection, the presence of a central venous line, haemodyalisis, Swan-Ganz and urinary catheters, draining tubes and mechanical ventilation. The presence of septic shock was defined by a systolic blood pressure ≤ 90 mmHg or a reduction of 70 mmHg in systolic blood pressure in hypertensive patients. The severity of illness at the time of bacteremia was classified by the Simplified Acute Physiology Score (SAPS) [12]. The presence of the following comorbid conditions was documented: cardiovascular diseases, solid or hematologic malignancies, neurological diseases, renal failure (indicated by a creatinine level >2.0 mg/dL or the requirement of dialysis), diabetes mellitus, hepatic dysfunction, chronic obstructive pulmonary disease, intravenous drug use, and HIV infection. Previous antibiotic treatment was defined as an antibiotic prescribed for at least 48 hours during the fifteen-day period prior to the onset of BSI [13].

Underlying diseases were classified according to the McCabe classification [14]. Nosocomial infections and sources of infection were defined according to Centers for Disease Control and Prevention (CDC) criteria [1]. Inadequate empiric antimicrobial treatment was defined as therapy administered within 24 hours after blood cultures were obtained that included the administration of an antimicrobial agent to which the *K. pneumoniae *isolate was resistant [16]. Antimicrobial agents were considered adequate if the organism was susceptible, except when cephalosporins were used for the treatment of ESBL infections [7]. A group of five infectious disease specialists responsible for BSI surveillance at our hospital assessed the adequacy of antimicrobial therapy (including the dosage and route of administration) for patients infected with *K. pneumoniae *BSI. We inform doctors (generally residents or fellows) about the blood culture results daily (in two periods, generally in the morning and in the afternoon). We based our adequacy of antimicrobial treatment for ESBL-producing *K. pneumoniae *on guidelines that were written and followed by these five infectious diseases specialists. Each case was reviewed by all five physicians, independently. Rarely, when there was any discordance among us, the final decision was made after further discussion with the majority deciding on the adequacy of antimicrobial therapy. Mortality was defined as death from any cause within 15 days from the date of the first positive blood culture for *K. pneumoniae*.

### Microbiological methods

Blood cultures (consisting of a pair of blood culture bottles including aerobic and anaerobic resin-containing media) obtained from adult patients were processed using the BACTEC^® ^9240 blood culture system (Beckton Dickinson, USA). Organisms were identified according to routine bacteriological procedures. Susceptibility testing was performed by the disk diffusion method, following the National Committee for Clinical Laboratory Standards (NCCLS) recommendations [17]. *K. pneumoniae *isolates were screened for the ESBL phenotype according to the NCCLS guidelines [18]. The ESBL phenotype was confirmed using the E-test ESBL (AB BIODISK, Solna, Sweden) [19].

### Statistical analysis

The association of variables was compared by the use of the χ^2 ^or Fisher's exact tests as appropriate. Significance probabilities (p values) were defined for entry and removal of variables in the logistic regression model: p value <0.05 (deaths from the univariate analysis) and p value <0.10, respectively. All tests of significance were two tailed.

When colinearity existed between two variables, only the one that had the greatest clinical relevance associated with mortality within 15 days was included in the multivariate analysis. Odds ratios were calculated for independent variables associated with 15-day mortality. The association of independent variables was expressed as odds ratios with 95% confidence intervals. A p value of <0.05 was considered statistically significant. All statistical calculations were performed using SPSS for MS Windows software (SPSS 11.0 for Windows).

## Results

During the study, 115 patients with *K. pneumoniae *BSI isolates were identified, of whom 108 met criteria for inclusion. ESBL-producing *K. pneumoniae *was detected in 56 of 108 patients (51%). We excluded two patients with pseudobacteremia by *K. pneumoniae *and five patients were excluded because the data in medical records were incomplete.

The average age was 27.3 years (range: 0–78 years). Forty-seven patients (43.5%) were less than 20 years. The most frequent diagnoses responsible for hospitalization were solid and hematologic malignancies (21.3%), abdominal diseases (14.8%), neonatal diseases (premature, congenital and genetic anomalies) (13.9%), renal failure (11%) and respiratory infections (10%). The most frequent sources of infection were pulmonary (38%), abdominal (26%), central venous catheter (14%) and urinary (11%). The median duration of hospitalization was 15.5 days. BSI occurred in 15.7%, 10.2%, and 11.1% in the first, second and third week of hospitalization, respectively. Most BSI (63.0%) occurred after 21 days of hospitalization, which can be seen in Table 1. The antibiotic therapy was considered adequate in 63.9% (69/108) of the cases and inadequate in 36.1% (39/108).

**Table 1:** Demographic characteristics of 108 patients with BSI due to *Klebsiella pneumoniae*.

**VARIABLES**	**N**	**%**
**Sex**		
Male	53	49.1
Female	55	50.9
**Age (years)**		
<1	31	28.7
1–10	11	10.2
11–20	5	4.6
21–60	42	38.9
>60	19	17.6
**Diagnosis**		
Neoplasm	23	21.3
Gastrointestinal disease	16	14.8
Neonatal diseases	15	13.9
Neurologic disease	12	11.1
Renal failure	12	11.1
Pulmonary infection	11	10.2
Cardiovascular disease	7	6.5
Others	7	6.5
Trauma	5	4.6
**Site of infection**		
Respiratory	41	38.0
Abdominal	28	25.9
Catheter	15	13.9
Urinary	12	11.1
Others	6	5.6
Central nervous system	4	3.7
Wound	2	1.9
**LOS prior to bacteremia (days)**		
< = 2	5	4.6
3–7	12	11.1
8–14	11	10.2
14–21	12	11.1
>21	68	63.0

LOS = Length of hospital stay

The proportions for the different variables in the two groups of patients in stratified by ESBL production are listed in Table 2. There were no age (<1 year or >60 years) or gender differences between the two groups (p = 0.693, p = 0.561 and p = 0.339, respectively). A higher proportion of patients with ESBL-producing strains were in intensive care units and had central venous catheters (p = 0.031 and p = 0.044, respectively). There was also a trend towards more than 10 days of hospitalization in patients with ESBL-producing strains when compared to patients with non-ESBL-producing strains BSI (p = 0.051). There was no relevant difference in other therapeutic procedures (pulmonary artery catheter, hemodialysis, urinary catheter, drainage tubes and the use of any class of antimicrobials) prior to *Klebsiella *BSI between the two groups. However, case patients were significantly more likely to need mechanical ventilation (p = 0.013). No statistically significant difference was observed for the McCabe classification, number of comorbidities, or sources of infection between the two groups (p = 0.623, p = 0.662 and p = 0.343, respectively). Septic shock was present in approximately one-quarter of the cases (p = 0.501) and thrombocytopenia (<80.000/mm^3^) was present in two-quarters of the cases (p = 0.438). Inadequate antimicrobial therapy was more common prescribed for case patients (41.1% vs. 30.8%), however this was not statistically significant (p = 0.265). Mortality at 15 days was more common in cases than in controls (p = 0.042). No time trends in mortality were observed (p = 0.601).

**Table 2:** Characteristics of patients according to ESBL-producing *K. pneumoniae *BSI from January 1996 to May 2001.

**Variables**	**ESBL (n = 56)**		**Non-ESBL (n = 52)**		
	**N**	**%**	**N**	**%**	***P***
**AGE (infants)**					
<1 year	17	54.8	14	45.2	0.693
≥ 1 year	39	50.6	38	49.4	
**AGE (elderly)**					
>60 years	11	57.9	8	42.1	0.561
< = 60 years	45	50.6	44	49.4	
**SEX**					
Male	25	47.2	28	52.8	0.339
Female	31	56.4	24	43.6	
**HOSPITAL LOCATION**					
ICU	31	63.3	18	36.7	0.031
Other units	25	42.4	34	57.6	
**HOSPITAL STAY (days)**					
>10 days	50	56.2	39	43.8	0.051
< = 10 days	6	31.6	13	68.4	
**CENTRAL VENOUS CATHETER**					
0	29	44.6	36	55.4	0.044
1	23	60.5	15	39.5	
≥ 2	4	80.0	1	20.0	
**SWAN-GANZ**					
Yes	2	66.7	1	33.3	1.000
No	54	51.4	51	48.6	
**HEMODIALYSIS**					
Yes	4	66.7	2	33.3	0.680
No	52	51.0	50	49.0	
**URINARY CATHETER**					
Yes	11	68.8	5	31.3	0.143
No	45	48.9	47	54.0	
**MECHANICAL VENTILATION**					
Yes	16	76.2	5	23.8	0.013
No	40	46.0	47	54.0	
**DRAINAGE TUBES**					
Yes	5	83.3	1	16.7	0.207
No	51	50.0	51	50.0	
**PREVIOUS ANTIBIOTICS**					
0	15	31.3	33	68.8	0.276
1	17	73.9	6	26.1	
2	14	56.0	11	44.0	
≥ 3	10	83.3	2	16.7	
**McCabe CLASSIFICATION**					
Rapidly fatal	9	52.9	8	47.1	0.623
Potentially fatal	6	37.5	10	62.5	
Non fatal	41	54.7	34	45.3	
**NUMBER OF COMORBIDITIES**					
None	19	51.4	18	48.6	0.662
1	20	47.6	22	52.4	
2	14	58.3	10	41.7	
3	3	75.0	1	25.0	
4	0	-	1	100.0	
**SOURCE OF BSI**					
Respiratory	23	56.1	18	43.9	0.343
Abdominal	12	42.9	16	57.1	
Urinary	8	66.7	4	33.3	
Catheter	6	40.0	9	60.0	
CNS	3	75.0	1	25.0	
Wound	2	100.0	0	-	
Abdominal	12	42.9	16	57.1	
Others	2	33.3	4	66.7	
**SEPTIC SHOCK**					
Yes	10	45.5	12	54.5	0.501
No	46	53.5	40	46.5	
**PLATELETS**					
≥ 80,000/mm^3^	31	50.8	30	49.2	0.438
<80,000/mm^3^	15	32.6	10	25.0	
**ANTIBIOTIC THERAPY**					
Adequate	33	47.8	36	52.2	0.265
Inadequate	23	41.1	16	30.8	
**15 DAY-MORTALITY**					
Yes	18	69.2	8	30.8	0.042
No	38	46.3	44	53.7	
**TIME TRENDS IN MORTALITY**					
1996–1998	21	55.3	17	44.7	0.601
1999–2001	35	50.0	35	50.0	

Mortality up to fifteen days after the BSI occurred in 24.1% (26/108) of the patients. The 30-day mortality and in-hospital mortality was 31.5% (34/108) and 37.0% (40/108), respectively. It was also observed that 15-day mortality was higher in patients hospitalized in intensive care units (p = 0.019), those with urinary catheters (p = 0.009), those mechanically ventilated (p = 0.001), and those with drainage tubes (p = 0.012).

Death in patients with potentially fatal or non-fatal underlying disease according to the MacCabe classification was 52.9% and 18.7%, respectively (p = 0.01). Patients with a SAPS score >40 (at the time of bacteremia) had a higher mortality rate than patients with a SAPS score ≤ 40, (63.3% vs. 26.2%, respectively, p = 0.014). The mortality in patients with shock was 45.5%, whereas in patients not requiring treatment with vasopressor drugs it was only 18.6% (p = 0.009). Platelet counts lower than 80,000/mm^3 ^were also associated with high mortality. The presence of more than two comorbidities was related to high mortality when compared to patients without any or with one comorbidity (48.2% vs. 15.2%, p = 0.003). Patients who received two or more antibiotics for more than 48 hours for the fifteen-day period before the diagnosis of BSI had higher than in those who received only one or no antibiotic before BSI (43.2% vs. 14.1%, p = 0.004).

In the subset of patients receiving adequate antibiotic therapy, higher mortality occurred in those acquiring ESBL-producing *K. pneumoniae *(36.4% vs. 11.1%, p = 0.013). On the other hand, in patients who received inadequate antibiotic therapy, there was no difference in 15-day mortality when patients with *K. pneumoniae *BSI with or without ESBL production were compared (26.1% vs. 25.0%, respectively, p = 1.0). Variables found to be statistically significant for 15-day mortality in BSI by univariate analysis were selected for multiple logistic regression. The following variables were independently related to death (table 3): mechanical ventilation (OR = 5.27, IC 95% = 1.9–14.6, p = 0.001), two or more comorbidities, (OR = 2.39, IC 95% = 1.3–4.2, p = 0.003), more than two antibiotics used before bacteremia (OR = 2.30, IC 95% = 1.2–4.3, p = 0.01), and the presence of a rapidly fatal disease (OR = 2.14, IC 95% = 1.1–4.2, p = 0.025).

**Table 3:** Independent predictors for 15-day mortality in BSI by *Klebsiella pneumoniae *BSI.

**Variables**	**OR**	**IC 95%**	**p**
Mechanical ventilation	5.27	1.9 – 14.6	0.001
Greater than two comorbidities	2.39	1.3 – 4.2	0.003
Greater than two antibiotics	2.30	1.2 – 4.3	0.010
Rapidly fatal disease	2.14	1.1 – 4.2	0.025

## Discussion

Of 108 patients with *K. pneumoniae *BSI, a high proportion produced ESBL (51.8%). It is one of the highest ESBL infection prevalence described in the literature comparing with Canada (5%), United States (8%) Europe (23%) and Pacific western region (25%) [8]. Because of the high incidence of this infection, we decided to investigate if the mortality is increased in *K. pneumoniae *strains producing ESBL. It is important to mention that before our study, Gales et al. [3] studied 72 strains of *K. pneumoniae *between January 1995 and October 1996 in the same institution, of which 39% were ESBL producers. However, these authors analyzed strains that were found in infections from many sources. Curiously, in our study, the frequency of occurrence of ESBL-producing *K. pneumoniae *was even higher than that observed by Gales et al. A possible explanation for this fact is that there was a dissemination of ESBL-producing strains over the years in our institution. However, the limitations of our study should be acknowledged. First, we do not have information regarding the specific situation in Brazil, such as genotyping results or type of ESBL. Only case reports were described [20,21]. Second, we performed a retrospective cohort study instead of a case-control study because it was not our objective to establish the risk factors for the acquisition of ESBL infection. Third, we did not observe a statistically significant difference in mortality between the ESBL producing *K. pneumoniae *and non- ESBL producing *K. pneumoniae *BSI. The small number of cases could lead to a type II error. And fourth we did not evaluate co-infections or recent infections with other nosocomial bacteria evaluated as predictors of mortality.

The 15-day mortality of *K. pneumoniae *BSI was 24.1%, and 69.2% in patients with ESBL producing strains. Hansen et al., in Denmark, reported similar mortality rates in *Klebsiella *bacteremia [22]. In contrast, Menashe et al., in Israel, reported a mortality rate of 43.6% with 50% of isolates producing ESBL [23]. By univariate analysis, we identified other variables associated with mortality, such as ICU stay, urinary catheter, mechanical ventilation, fatal underlying disease, two or more comorbidities, a SAPS score >40, shock, thrombocytopenia (<80,000/mm^3^) and previous use of two or more antibiotics, which have been demonstrated in previous studies [22-25]. To characterize the severity of the patients' conditions in this study, in addition to the MacCabe classification, we utilized the SAPS-II classification (Simplified Acute Physiology Score) at the time of bacteremia. We decided to use this classification method instead of the APACHE II system used in intensive care therapy, because it provides better-defined criteria. However, the data are collected in the first twenty-four hours of admission and is applicable only for patients in intensive care. In addition, the data do not present the seriousness of the patient's condition during the hospitalization period. Through a univariate analysis, we found that patients with a SAPS-II score >40 have a higher chance of mortality when compared with patients with a SAPS-II score ≤ 40 (p = 0.014).

Multivariate analysis identified that the most important risk factor for death was the requirement for mechanical ventilation. The respiratory tract was the source of BSI in 38% of patients. Fifty-two percent of the patients required mechanical ventilation. The number of comorbidities (more than two) and the presence of fatal underlying disease were the variables associated with death during the fifteen days following *K. pneumoniae *BSI, demonstrating the importance of the patient's underlying diseases. The same results were obtained by Garrouste-Orgeas et al. in their study. These authors observed that the outcome was influenced by the severity of the underlying host conditions, particularly with patients requiring mechanical ventilation [26]. The prior use of two or more antibiotics was independently associated with death, suggesting a possible selection of resistant antimicrobial strains, such as ESBL-producing strains [25].

By multivariate analysis, previous studies [23,24,27,28] have reported no increase in the mortality rate of infections caused by this resistant microorganism; these studies identified the clinical implications of extended-spectrum beta-lactamase (ESBL) production, not only for *Klebsiella pneumoniae*, but also for *E. coli*. Many studies have demonstrated that inadequate antibiotic therapy is related to an increase in the mortality rate [7,15,16,29]. The adequacy of antibiotic therapy was similar when we compared ESBL and non-ESBL producing strains of *K. pneumoniae*. We considered as inadequate the use of cephalosporins for all ESBL- producing *K. pneumoniae *BSIs cases in the hospital [7]. Another important consideration is that our institution has a high rate not only of ESBL-producing *K. pneumoniae *but also ceftazidime-resistant *Pseudomonas aeruginosa *and *Acinetobacter baumannii*. It certainly changed our clinical practice over time and contributed to a better appropriate antimicrobial treatment for this types of infections. We believe that it limits our ability to generalize these findings to hospitals with lower prevalence of ESBL-producing *K. pneumoniae*. In our institution the empiric therapy for gram-negative infections is carbapenems. Conversely, Kim et al. had more cases of ESBL-producing *K. pneumoniae *than non-ESBL-producing *K. pneumoniae *receiving inappropriate antibiotic therapy before culture results were reported (54.5 vs. 3.4%; P = 0.001). In our study, inadequate empirical therapy showed no statistically significant difference in relation to death regardless of ESBL production (p = 0.117). It likely reflects the severity of the patient's underlying diseases. However, in the study of Kim et al., which evaluated 19 cases of ESBL-producing *K. pneumoniae *BSI, no significant difference in mortality was observed between patients who received appropriate empiric antibiotic therapy and those who did not (26.3 vs. 20.8%; P = 0.67). One the other hand, Hyle et al. observed that inadequate initial antimicrobial therapy was an independent risk factor for mortality in ESBL-producing *K. pneumoniae *BSIs [31].

The mortality rate of BSI remains high, despite adequate antibiotic treatment and intensive care measures. In our study, an increase in mortality in hospital-acquired BSI was related to the need for mechanical ventilation, rapidly fatal disease, more than two comorbidities and the use of more than two antibiotics before infection. Additional studies should be carried out to confirm the relationship between high mortality and ESBL-producing *K. pneumoniae*.

## Pre-publication history

The pre-publication history for this paper can be accessed here:


